# Integrated analysis of three newly sequenced fern chloroplast genomes: Genome structure and comparative analysis

**DOI:** 10.1002/ece3.7350

**Published:** 2021-03-18

**Authors:** Ruifeng Fan, Wei Ma, Shilei Liu, Qingyang Huang

**Affiliations:** ^1^ School of Pharmacy Heilongjiang University of Chinese Medicine Harbin China; ^2^ Experimental Teaching & Practical Training Center Heilongjiang University of Chinese Medicine Harbin China; ^3^ Department of Ecology Institute of Natural Resources and Ecology Heilongjiang Academy of Science Harbin China

**Keywords:** Chloroplast genome, Fern, Phylogenetic tree, RNA editing, Simple sequence repeat

## Abstract

**Background:**

Some ferns have medicinal properties and are used in therapeutic interventions. However, the classification and phylogenetic relationships of ferns remain incompletely reported. Considering that chloroplast genomes provide ideal information for species identification and evolution, in this study, three unpublished and one published ferns were sequenced and compared with other ferns to obtain comprehensive information on their classification and evolution.

**Materials and Methods:**

The complete chloroplast genomes of *Dryopteris goeringiana* (Kunze) Koidz, *D. crassirhizoma* Nakai, *Athyrium brevifrons* Nakai ex Kitagawa, and *Polystichum tripteron* (Kunze) Presl were sequenced using the Illumina HiSeq 4,000 platform. Simple sequence repeats (SSRs), nucleotide diversity analysis, and RNA editing were investigated in all four species. Genome comparison and inverted repeats (IR) boundary expansion and contraction analyses were also performed. The relationships among the ferns were studied by phylogenetic analysis based on the whole chloroplast genomes.

**Results:**

The whole chloroplast genomes ranged from 148,539 to 151,341 bp in size and exhibited typical quadripartite structures. Ten highly variable loci with parsimony informative (*Pi*) values of > 0.02 were identified. A total of 75–108 SSRs were identified, and only six SSRs were present in all four ferns. The SSRs contained a higher number of A + T than G + C bases. C‐to‐U conversion was the most common type of RNA editing event. Genome comparison analysis revealed that single‐copy regions were more highly conserved than IR regions. IR boundary expansion and contraction varied among the four ferns. Phylogenetic analysis showed that species in the same genus tended to cluster together with and had relatively close relationships.

**Conclusion:**

The results provide valuable information on fern chloroplast genomes that will be useful to identify and classify ferns, and study their phylogenetic relationships and evolution.

## Graphical abstract

1. Study reported three newly sequenced chloroplast genomes of ferns.
2. Study provided more information on understanding evolution and phylogenetic of ferns.

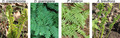

## 1 INTRODUCTION

Ferns are the most evolved of the spore‐forming plants, and some have medicinal properties (Chen et al., 2007). The genus *Dryopteris* (Dryopteridaceae, comprising 225–300 species) is considered ideal for studying diversification, hybridization, and polyploidy in ferns (Sessa et al., 2012). *D. goeringiana* and *D. crassirhizoma*, which originated in the northeast region of China are distributed across Russia, Japan, and North Korea. The rhizome and petiole residues of *D. crassirhizoma* are used in traditional Chinese herbal medicine to eliminate heat and toxins, promote blood circulation, and treat blood stasis (Z. Zhao et al., 2007). *Polystichum* (Dryopteridaceae) is one of the most abundant genera of ferns and commonly occurs in lowlands and montane to alpine areas (Zhang, 2012); it contains 500 species, with 208 species known in China (Zhang & Barrington). *Athyrium Roth* (Athyriaceae), the lady‐fern genus, contains approximately 220 described species (Ran Wei & Zhang, 2016). *Athyrium brevifrons* is often used as a wild vegetable in northeastern China because of its high nutritional value. Because only a small proportion of ferns have been identified and classified, additional studies are needed.

With the development of next‐generation sequencing (NGS) technology, the details of the most subtle nuclear gene components in eukaryotic cells have become clearer, and the study of cytoplasmic organelle genomes has also been facilitated in a more straightforward and time‐saving way (Ruiz‐Ruano et al., 2018). This is especially true for chloroplasts, which are involved in many biochemical metabolism processes, including amino acid, sugar, lipid, vitamin, starch, and pigment synthesis; sulfate reduction; and nitrogen. Most chloroplast converts light energy into chemical energy via photosynthesis, making chloroplasts indispensable for plants (Bausher et al., 2006; Jarvis & Soll, 2001; Leister, 2003). Compared with nuclear genomes, chloroplast genomes are more highly conserved in terms of gene order, gene content, and substitution rate (Green, 2011; Helena, 2004; Ruhlman & Jansen, 2014; Wolfe et al., 1987; J. H. Xu et al., 2015). Chloroplast genomes have a typically circular structure with one large single‐copy (LSC) region, one short single‐copy (SSC) region, and two inverted repeat (IR) regions, ranging from 120 to 170 kilobases in length (Downie & Palmer, 1992). Owing to the absence of recombination and maternal transmission, the chloroplast genomes are helpful for tracing source populations (McCauley et al., 1996; Small et al., 2004). They have become a valuable and ideal resource for species identification, population genetics, plant phylogenetics, and genetic engineering considering their similar structures, highly conserved sequences, and stable maternal heredity (Nock et al., 2014). However, gain and loss of genes, gene content duplication, and gene order rearrangements appear to be phylogenetically and species informative (Bausher et al., 2006; Green, 2011; J. H. Xu et al., 2015).

An increasing number of chloroplast genomes have been reported in recent years, especially because NGS has become cheaper and faster. The chloroplast genomes of many plants have been sequenced, including those of bryophytes (M Park et al., 2018; P. Wolf & Karol, 2012), lycophytes (Guo et al., 2016; Tsuji et al., 2007), monophytes (Logacheva et al., 2017; Lu et al.,.., 2015; Ruiz‐Ruano et al., 2018; R. Wei et al., 2017; P. G. Wolf et al., 2011), and spermatophytes (Sun et al., 2016; J. H. Xu et al., 2015). As one of the largest group of vascular plants, approximately 2,129 species of ferns are present in China (Z. et al.,.., 2013), of which only 60 have been reported.

*D. crassirhizoma*, *D. goeringiana*, *A. brevifrons*, and *P. tripteron* studied in the present study are relatively distributed in Heilongjiang Province, China, and all have certain antibacterial effects or edible value. These ferns are also the research hotspots of domestic ferns. Comparing the differences between the chloroplast genomes through the genetic relationship at different levels provides theoretical support for further development and utilization. In the present study, the complete chloroplast genomes of *D. goeringiana*, *A. brevifrons*, and *P. tripteron* were sequenced for the first time. We performed comparisons of the genomes and IR boundary expansion and contraction. Simple sequence repeats (SSRs), highly variable loci, and RNA editing events were also investigated in the four ferns. A phylogenetic tree was constructed based on the chloroplast genomes of almost all ferns reported thus far. The present study was conducted to achieve the following objectives: a) to sequence and report the chloroplast genomes of *D. crassirhizoma*, *D. goeringiana*, *A. brevifrons*, and *P. tripteron*; b) to compare the chloroplast genomes of eight fern species to identify useful DNA barcodes for plant identification and evolution analysis; and c) to identify a more comprehensive phylogenetic relationship among ferns.

## 2 MATERIALS AND METHODS

### 2.1 Materials

**FIGURE 1:**
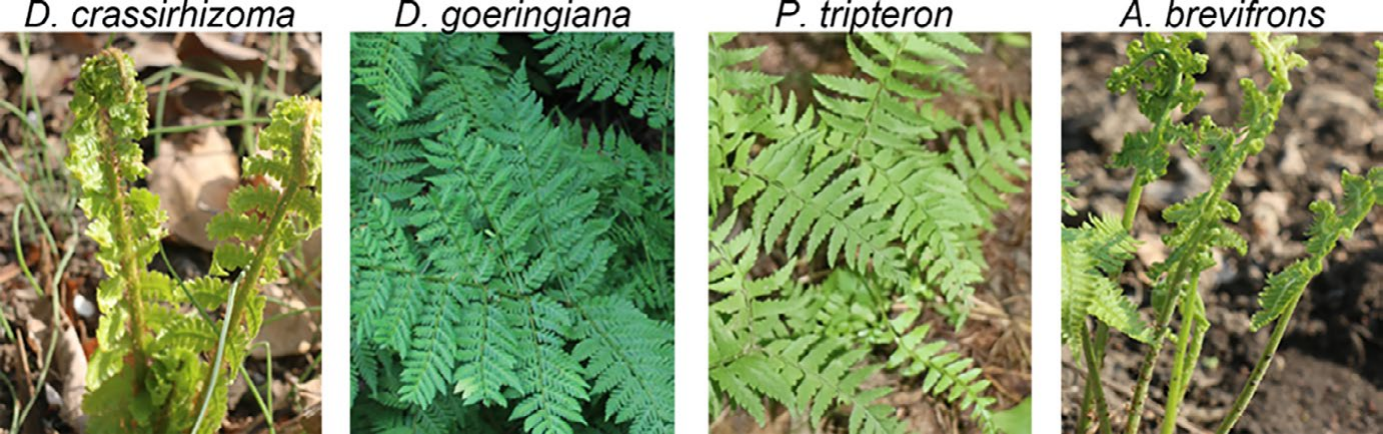
Morphological characteristics of *D. goeringiana*, *A. brevifrons*, *D. crassirhizoma*, and *P. tripteron*

Wild specimens of *D. crassirhizoma*, *A. brevifrons*, *D. goeringiana*, and *P. tripteron* were collected from Maoer Mountain, Maoershan Town, Shangzhi City, Heilongjiang Province (N 45°17′51.45′′, E127°36′00.03′′), China (Figure 1). The four species were identified by Ruifeng Fan from the Heilongjiang University of Chinese Medicine. Voucher specimens were deposited in the Northeast Agricultural University Herbarium with the collection numbers 2018–21 (*D. crassirhizoma*), 2018–22 (*A. brevifrons*), 2018–32 (*D. goeringiana*), and 2018–33 (*P. tripteron*).

### 2.2 Chloroplast DNA extraction and sequencing

Fresh leaves were collected, immersed in liquid nitrogen immediately, and stored at − 80℃ prior to DNA extraction. We isolated chloroplast DNA using an improved extraction method (McPherson et al., 2013) and evaluated its quality and quantity using a NanoDrop® 2000 spectrophotometer (Thermo Fisher Scientific, Wilmington, DE, USA) and a Qubit® 3.0 fluorometer (Invitrogen, Carlsbad, CA, USA), respectively. Samples that had a total amount of > 1 μg DNA and OD_260/280_ of 1.8–2.0 were used for library preparation.

Libraries were constructed with 1 μg of chloroplast DNA according to the Illumina TruSeq™ Nano DNA Sample Prep Kit (Illumina Inc, San Diego, USA) protocol. The libraries were sequenced using the Illumina HiSeq 4,000 platform (Biozeron Co., Ltd., China) (Borgström et al., 2011).

### 2.3 Genome assembly and annotation

Prior to assembly, low‐quality reads were removed using FastQC (http://www.bioinformatics.babraham.ac.uk/projects/fastqc/). Then, the chloroplast genomes were assembled in three steps as follows (Cronn et al., 2008): Clean reads were assembled into contigs using SOAP*denovo* 2.04 (Luo et al., 2012), clean reads were mapped to the contigs for assembly and optimization using SOAPGapCloser 1.12 (Q.‐Y. Zhao et al., 2011), and redundant sequences were removed.

Gene comparisons, predictions, and combinations were performed using Genewise (https://www.ebi.ac.uk/Tools/psa/genewise/), AUGUSTUS (http://bioinf.uni‐greifswald.de/augustus/), and EVidenceModeler version 1.1.1, respectively. Protein‐coding genes, tRNA genes, and rRNA genes were predicted using the DOGMA tool (Wyman et al., 2004). The assembled chloroplast genomes were then BLASTed against a series of databases, including Clusters of Orthologous Groups (Tatusov et al., 2003), Swiss‐Prot (Magrane, 2011), Gene Ontology (Ashburner et al., 2000), and Kyoto Encyclopedia of Genes and Genomes (Kanehisa et al., 2004; Minoru et al., 2006). Circular chloroplast genome maps of *D. crassirhizoma*, *A. brevifrons*, *D. goeringiana*, and *P. tripteron* were constructed using Organellar Genome DRAW 1.2 (Lohse et al., 2007).

### 2.4 SSR analysis

MIcroSAtellite identification tool (http://pgrc.ipk‐gatersleben.de/misa/) was used to detect the SSRs of *D. crassirhizoma*, *A. brevifrons*, *D. goeringiana*, and *P. tripteron*. The minimum number of repeats was set to eight, five, and four, for mononucleotide, dinucleotides, and trinucleotides, respectively, and to three for tetranucleotides, pentanucleotide, and hexanucleotides. The distance between two SSRs should be shorter than 100 bp. The SSR primers were designed using Primer3 (http://www.simgene.com/Primer3).

### 2.5 Nucleotide diversity analysis

We measured the parsimony informative (*Pi*) characters per‐site values to identify the most variable chloroplast genes using MAFFT 7.123b (http://mafft.cbrc.jp/alignment/software/) and Variscan 2.0 (http://www.ub.es/softevol/variscan). *Pi* values were calculated with a step size of 200 bp and a slide window of 300 bp. Loci with a *Pi* value of > 0.20 were considered as highly variable regions.

### 2.6 RNA editing

The RNA editing events were counted in four ferns.

### 2.7 Comparison analysis

Genome comparison analysis was conducted for *D. crassirhizoma*, *A. brevifrons*, *D. goeringiana*, and *P. tripteron* and for the additional ferns *Cyrtomium devexiscapulae*, *D. decipiens*, *Lepisorus clathratus*, and *Polypodium glycyrrhiza* using mVISTA (http://genome.lbl.gov/vista/mvista/about.shtml) in Shuffle‐LAGAN mode. We compared IR boundary expansion and contraction in all the above‐mentioned ferns, except *P. glycyrrhiza*.

### 2.8 Phylogenetic analysis

MAFFT v7.149 was used to align the cpDNAs sequences under default parameters (Katoh et al., 2005), and the alignment was trimmed by Gblocks_0.91b to remove low‐quality regions with the parameters: ‐t = d ‐b4 = 5 ‐b5 = h (Castresana, 2000). The maximum‐likelihood (ML) methods were performed for the genome‐wide phylogenetic analyses using PhyML 3.0 (Guindon et al., 2010), respectively. Nucleotide substitution model selection was estimated with jModelTest 2.1.10 (Darriba et al., 2012) and smart model selection in PhyML 3.0. The model general time‐reversible (GTR)+I + G was selected for ML analyses with 1,000 bootstrap replicates to calculate the bootstrap values of the topology. The results were treated with iTOL 3.4.3 (Letunic & Bork, 2016). *Adiantum capillus‐veneris* and *Myriopteris* (*Cheilanthes*) *lindheimeri* were included as outgroup species.

## 3 RESULTS

### 3.1 Chloroplast DNA sequencing and genomic features

Overall, 26,981,820–34,833,094 paired‐end (2 × 150 bp) raw reads were obtained from the four ferns (Table 1). The Q30 ranged from 89.06% to 91.52%.

**TABLE 1:** The summary of sequencing data and complete chloroplast genomes of *D. crassirhizoma*, *A. brevifrons*, *D. goeringiana*, and *P. tripteron*

Items	Species			
	*D. crassirhizoma*	*A. brevifrons*	*D. goeringiana*	*P. tripteron*
Sequencing				
Total Reads (Mb)	3.064	3.483	2.698	2.972
Total Bases (G)	4.627	5.260	4.074	4.488
Q30 (%)	89.06	89.07	90.97	91.52
Genome				
Total length (bp)	149,468	151,341	148,947	148,539
GC content (%)	43.19	43.76	43.12	42.40
LSC				
Length (bp)	82,504	82,459	82,384	82,799
Percentage (%)	55.20	54.49	55.31	55.74
SSC				
Length (bp)	21,600	21,708	21,623	21,660
Percentage (%)	14.45	14.34	14.52	14.58
IR				
Length (bp)	22,682	23,588	22,471	22,040
Percentage (%)	15.18	15.59	15.09	14.84
Protein‐coding genes				
Total gene number	89	89	89	89
Duplicated gene number	5	5	5	5
Single gene number	84	84	84	84
tRNA genes				
Total gene number	36	38	38	35
Duplicated gene number	3	5	6	4
Single gene number	33	33	32	31
rRNA genes				
Total gene number	8	8	8	8
Duplicated gene number	4	4	4	4
Single gene number	4	4	4	4

Note

LSC, large single copy; SSC, short single copy; IR, inverted repeats; tRNA, transfer RNA; rRNA, ribosomal RNA. *D. crassirhizoma*, *Dryopteris goeringiana* (Kunze) Koidz; *A. brevifrons*, *Athyrium brevifrons* Nakai ex Kitagawa; *D. goeringiana*, *Dryopteris crassirhizoma* Nakai; *P. tripteron*, *Polystichum tripteron* (Kunze) Presl.

**FIGURE 2:**
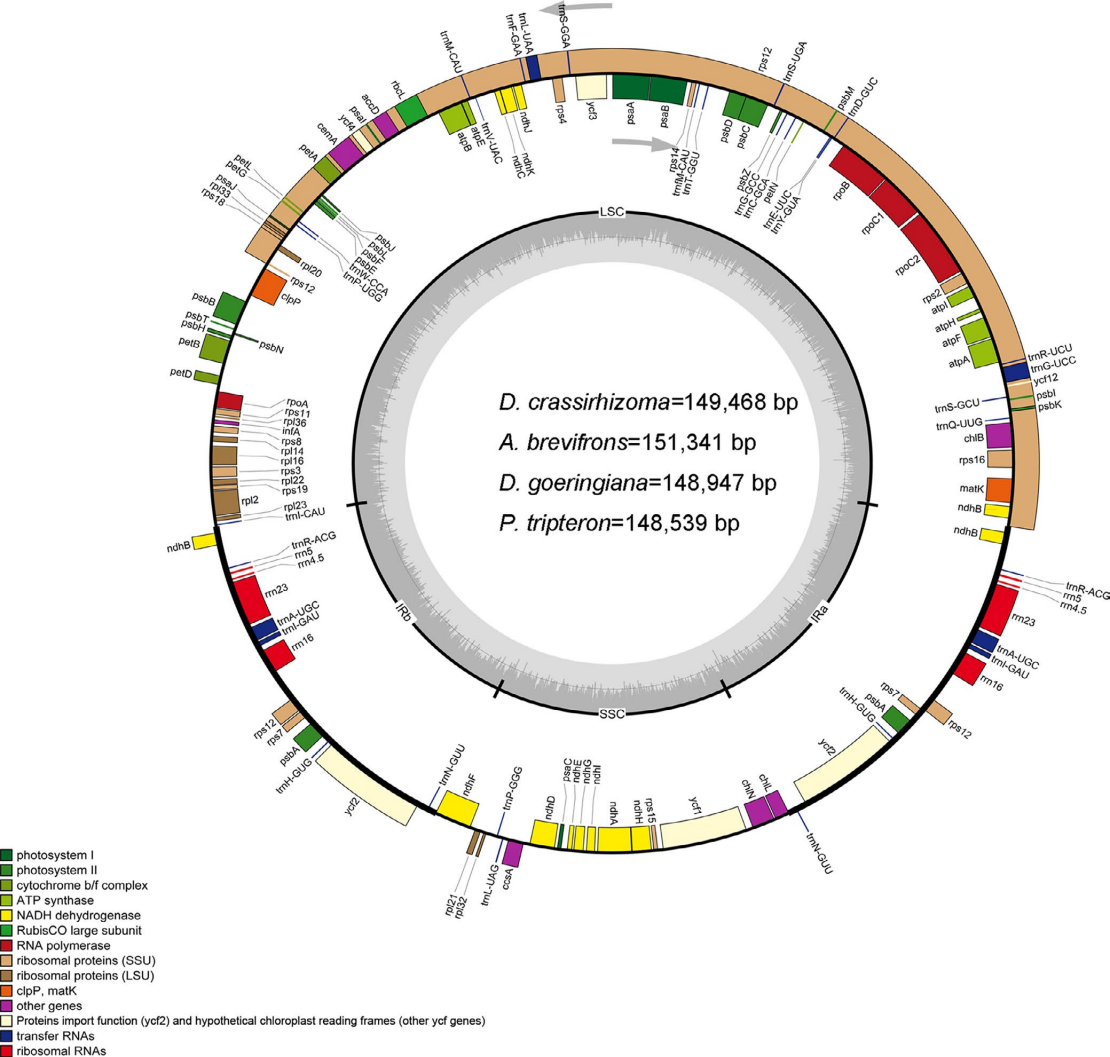
The chloroplast genome maps of *D. goeringiana*, *A. brevifrons*, *D. crassirhizoma*, and *P. tripteron*. Genes drawn inside the circle are transcribed clockwise, and those outside the circle are transcribed counterclockwise. The light gray inner circle corresponds to the A + T content, the dark gray to the G + C content. Genes belonging to different functional groups are shown in different colors

The genome size ranged from 149,468 bp (*D. crassirhizoma*) to 151,341 bp (*A. brevifrons*). The chloroplast genomes had a circular assembly and exhibited a typical quadripartite structure (Figure 2), including one LSC region (82,384–82,799 bp), one SSC region (21,600–21,708 bp), and two IR regions (22,040–22,682 bp) (Table 1). The overall G + C content was 42.40%–43.76%.

Each fern genome was composed of 89 protein‐coding genes, eight rRNA genes, and 35–38 tRNA genes. After removing the duplications, 84 protein‐coding genes, four rRNA genes, and 31–33 tRNA genes remained (Tables 1 and 2). The type and number of tRNA genes were distinct in the LSC, SSR, and IRs regions (Table 3). *trnN*‐*GUU* was present only in the SSC region of *A. brevifrons*. *trnI‐GAU* was absent only in the IRb region of *P. tripteron*. A total of 14 genes contained introns: 11 genes (*atpF*, *matK*, *ndhA*, *ndhB*, *petA*, *petB*, *petD*, *rpl16*, *rpl2*, *rpoC1*, *rpoB*, and *rps16*) contained one intron and three genes (*clpP*, *rps12*, and *ycf3*) contained two introns (Table 4). Notably, *matK* in *P. tripteron* contained two introns, whereas *matK* in the other three species contained only one intron. *rps12*, with one exon in the LSC region and the other two in the IR regions, was considered a trans‐spliced gene separated by two introns.

**TABLE 2:** The common gene list of *D. crassirhizoma*, *A. brevifrons*, *D. goeringiana*, and *P. tripteron*

Category	Gene names	
Photosynthesis	Subunits of photosystem I	*psaA psaB psaC psaI psaJ*
	Subunits of photosystem II	*psbA psbA‐D2 psbB psbC psbD psbE psbF psbH psbI psbJ psbK psbL psbM psbN psbT psbZ*
	Subunits of NADH dehydrogenase	*ndhA ndhB ndhB‐D2 ndhC ndhD ndhE ndhF ndhG ndhH ndhI ndhJ ndhK*
	Subunits of cytochrome b/f complex	*petA petB petD petG petL petN*
	Subunits of ATP synthase	*atpA atpB atpE atpF atpH atpI*
	Large subunit of Rubisco	*rbcL*
Self‐replication	Large subunits of ribosome	*rpl14 rpl16 rpl2 rpl20 rpl21 rpl22 rpl23 rpl32 rpl33 rpl36*
	Small subunits of ribosome	*rps11 rps12 rps12‐D2 rps14 rps15 rps16 rps18 rps19 rps2 rps3 rps4 rps7 rps7‐D2 rps8*
	DNA‐dependent RNA polymerase	*rpoA rpoB rpoC1 rpoC2*
	Ribosomal RNAs	*rrn16 rrn23 rrn4.5 rrn5*
Other genes	Maturase	*matK*
	Protease	*clpP*
	Envelope membrane protein	*cemA*
	Acetyl‐CoA carboxylase	*accD*
	C‐type cytochrome synthesis gene	*ccsA*
	Translation initiation factor	*infA*
	Proteins import function	*ycf2 ycf2‐D2*
	Proteins with unknown functions	*ycf1 ycf12 ycf3 ycf4*

Note

tRNA varied in four species and are listed in Table 3.

**TABLE 3:** List of genes distributed in different regions of *D. crassirhizoma*, *A. brevifrons*, *D. goeringiana*, and *P. tripteron* chloroplast genomes

Items	species			
	*D. crassirhizoma*	*A. brevifrons*	*D. goeringiana*	*P. tripteron*
Common tRNA genes	trnA‐UGC trnC‐GCA trnD‐GUC trnE‐UUC trnF‐GAA trnG‐GCC trnH‐GUG trnI‐CAU trnL‐UAA trnL‐UAG trnM‐CAU trnN‐GUU trnP‐GGG trnP‐UGG trnQ‐UUG trnR‐ACG trnR‐UCU trnS‐GCU trnS‐GGA trnS‐UGA trnT‐GGU trnV‐UAC trnW‐CCA trnY‐GUA trnfM‐CAU			
varied tRNA genes in LSC region	trnG‐UCC	trnG‐UCC−1, trnG‐UCC−2	trnG‐UCC−1, trnG‐UCC−2	trnG‐UCC−1, trnG‐UCC−2
varied tRNA genes in SSC region	–	trnN‐GUU	–	–
varied tRNA genes in IR region	trnN‐GUU × 2 trnI‐GAU × 2	trnI‐GAU × 2	trnN‐GUU × 2 trnI‐GAU × 2	trnN‐GUU × 2

Note

– represents no gene. LSC, large single‐copy; SSC, short single‐copy; IR, inverted repeats; tRNA, transfer RNA. *D. crassirhizoma*, *Dryopteris goeringiana* (Kunze) Koidz; *A. brevifrons*, *Athyrium brevifrons* Nakai ex Kitagawa; *D. goeringiana*, *Dryopteris crassirhizoma* Nakai; *P. tripteron*, *Polystichum tripteron* (Kunze) Presl.

**TABLE 4:** The length of introns in *D. crassirhizoma*, *A. brevifrons*, *D. goeringiana*, and *P. tripteron*

Introns	*D. goeringiana*	*A. brevifrons*	*D. crassirhizoma*	*P. tripteron*
*matK*‐CDS2_*matK*‐CDS1	12	12	12	12
*matK*‐CDS3_*matK*‐CDS2	–	–	–	482
*rps16*‐CDS2_*rps16*‐CDS1	822	797	807	799
*atpF*‐CDS2_*atpF*‐CDS1	728	704	728	720
*rpoC1*‐CDS2_*rpoC1*‐CDS1	695	701	696	691
*ycf3*‐CDS3_*ycf3*‐CDS2	625	625	626	620
*ycf3*‐CDS2_*ycf3*‐CDS1	731	729	731	731
*petA*‐CDS1_*petA*‐CDS2	12	12	12	12
*clpP*‐CDS3_*clpP*‐CDS2	555	585	555	572
*clpP*‐CDS2_*clpP*‐CDS1	705	701	705	717
*petB*‐CDS1_*petB*‐CDS2	788	795	789	818
*petD*‐CDS1_*petD*‐CDS2	89	638	89	651
*rpl16*‐CDS2_*rpl16*‐CDS1	768	758	766	775
*rpl2*‐CDS2_*rpl2*‐CDS1	723	724	725	746
*rps12*‐D2‐CDS2_*rps12*‐D2‐CDS3	577	578	577	577
*ndhA*‐CDS2_*ndhA*‐CDS1	943	962	944	978
*rps12*‐CDS3_*rps12*‐CDS2	577	578	577	577
Total length (bp)	9,350	9,899	9,339	10,478

Note

*D. crassirhizoma*, *Dryopteris goeringiana* (Kunze) Koidz; *A. brevifrons*, *Athyrium brevifrons* Nakai ex Kitagawa; *D. goeringiana*, *Dryopteris crassirhizoma* Nakai; *P. tripteron*, *Polystichum tripteron* (Kunze) Presl. CDS, coding sequences.

### 3.2 SSR analysis

**FIGURE 3:**
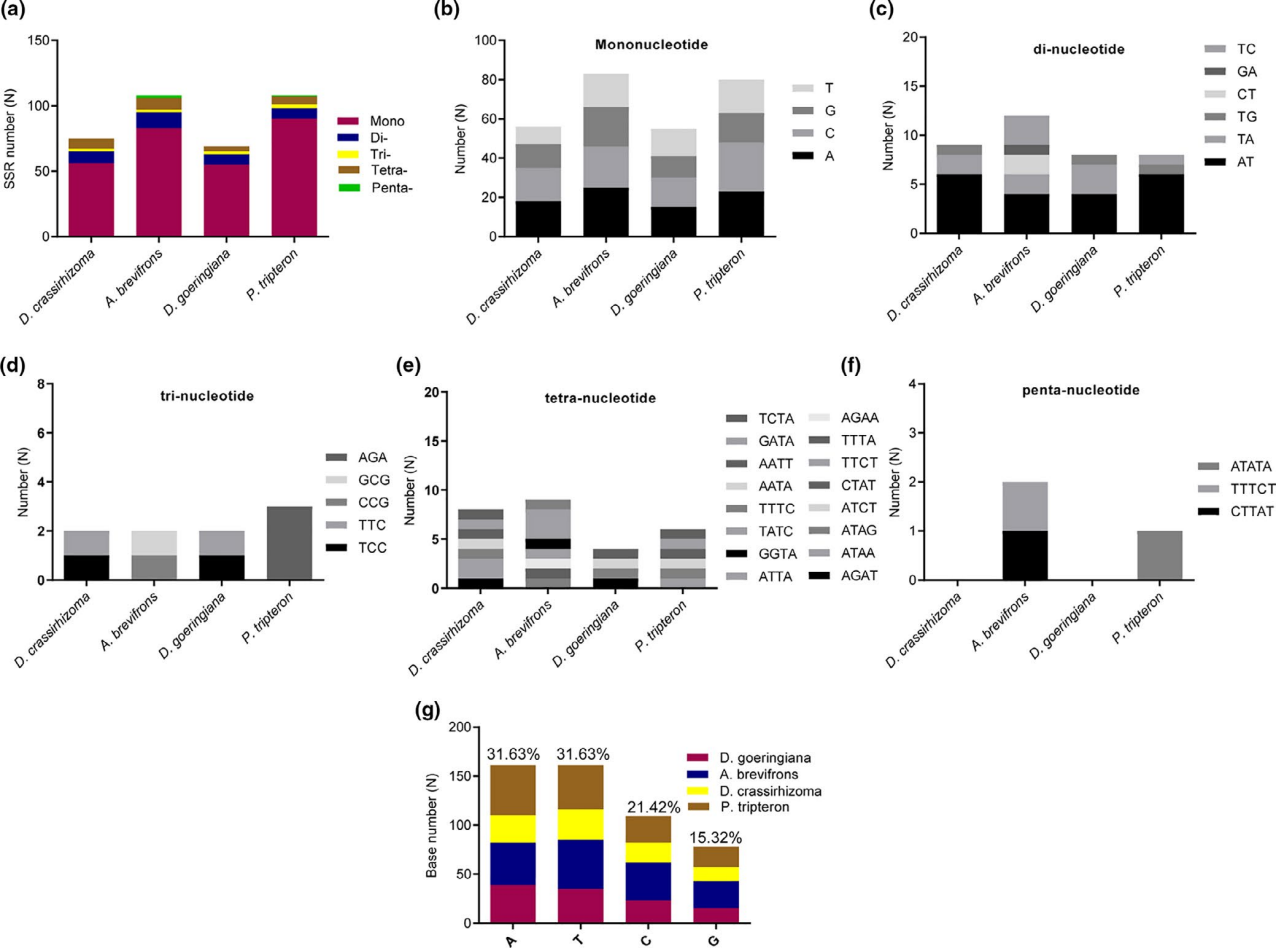
SSRs analyses in the four fern chloroplast genomes. (A) the number of different SSR types detected in the four genomes; (B) the number and types of identified mononucleotide SSR in the four genomes; (C) the number and types of di‐nucleotides SSRs in the four genomes; (D) the number and types of trinucleotides SSR in the four genomes; (E) the number and types of tetranucleotides SSR in the four genomes; (F) the number and types of pentanucleotide SSR in the four genomes; (G) the contents of A, T, C, and G in four ferns. SSR, simple sequence repeats

Five types of SSRs were identified, including mononucleotides, dinucleotides, trinucleotides, tetranucleotides, and pentanucleotides, with a total of 75–108 SSRs in the four species (Figure 3a and Table S1). There were 55–90 mononucleotides, 8–12 dinucleotides, 2–3 trinucleotides, 8–9 tetranucleotides, and 0–2 pentanucleotides (Figure 3b‐f). The types of SSRs of *D. crassirhizoma* were similar to those of *D. goeringiana*, but were more varied, with (ATAA)2, (ATCT)1, and (TTTA)1 also detected (Figure 3e and Table S1). Only six types of SSRs were simultaneously present in the four ferns: A, C, G, T, AT, and AGAT. The SSRs were composed of a higher number of A + T (63.26%) bases than G + C bases (36.74%; Figure 3g).

### 3.3 Nucleotide diversity analysis

**FIGURE 4:**
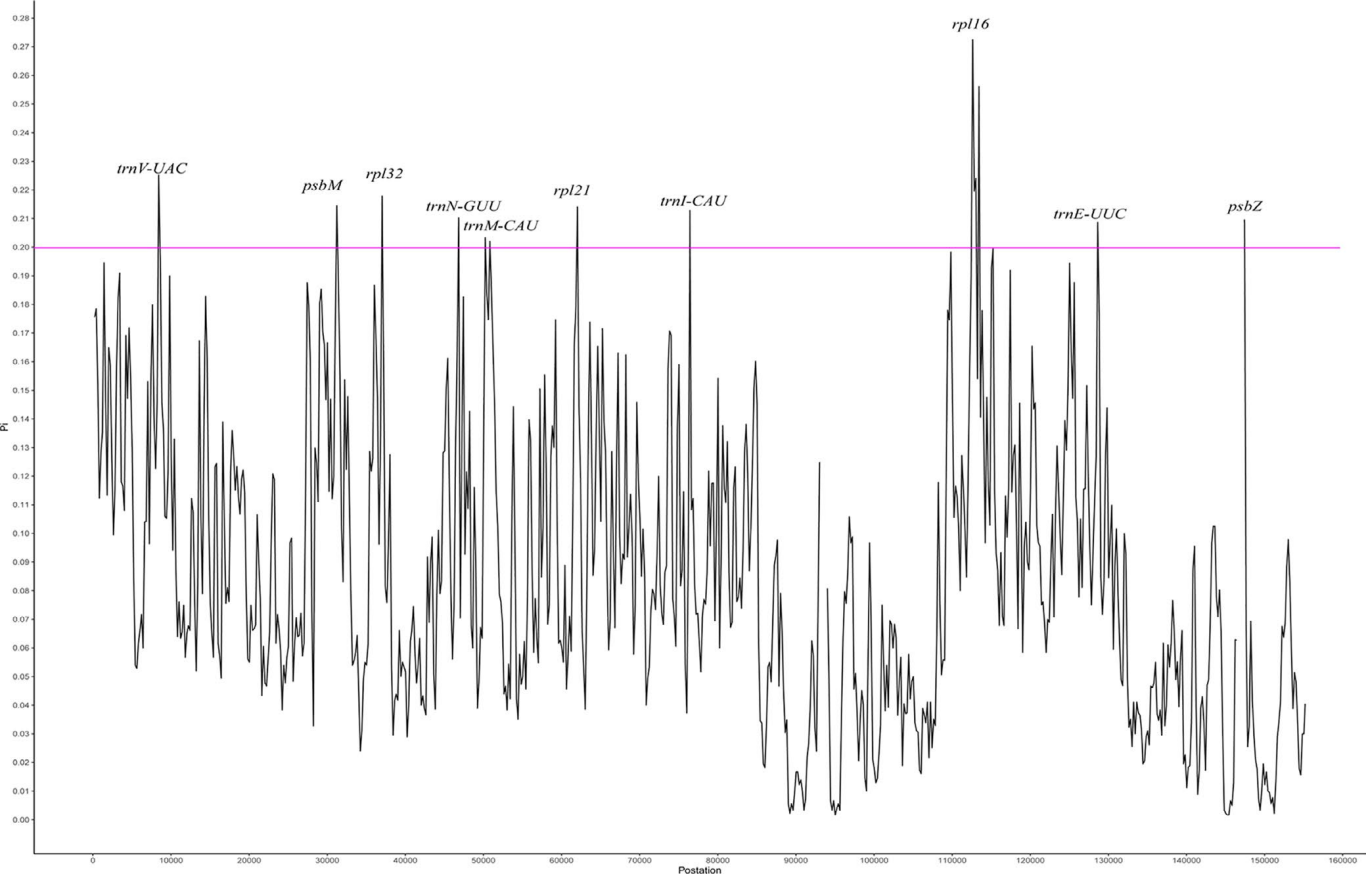
Comparative analysis of the nucleotide variability by *Pi* values within four fern species. *Pi* value of > 0.20 was wrote down. X‐axis: the position of the genome; Y‐axis: *Pi* value. *Pi*, polymorphism information

The chloroplast genome contains numerous variable nucleotides, which are usually recognized as valuable DNA barcoding regions for resolving closely related species or genera. In the present study, variable loci were identified in the four species, with *Pi* values ranging from 0.0000 to 0.2778 (*rpl16*) (Figure 4 and Table S2). Ten loci with a *Pi* value of > 0.2, which were mainly located at SC regions, were considered highly variable loci: *trnM‐CAU*, *trnE‐UUC*, *psbZ*, *trnN‐GUU*, *trnI‐CAU*, *rpl21*, *psbM*, *rpl32*, *trnV‐UAC*, and *rpl16*.

### 3.4 RNA editing

RNA editing is defined as the post‐transcriptional modification of precursor RNAs to alter their nucleotide sequences through the insertion and deletion, or specific substitution of nucleotides to introduce or remove start or stop codons or yield functional RNA species (Tsudzuki et al., 2001). In the present study, a total of 268 RNA editing events were identified in the four chloroplast genomes: 85 in *D. crassirhizoma*, 55 in *A. brevifrons*, 50 in *D. goeringiana*, and 78 in *P. tripteron*. C‐to‐U conversion (120 events, 44.8%) was the most prevalent RNA editing event, followed by U‐to‐C (103 events, 38.4%), A‐to‐G (36 events, 13.4%), and G‐to‐A (9 events, 3.4%).

### 3.5 Genome comparison

**FIGURE 5:**
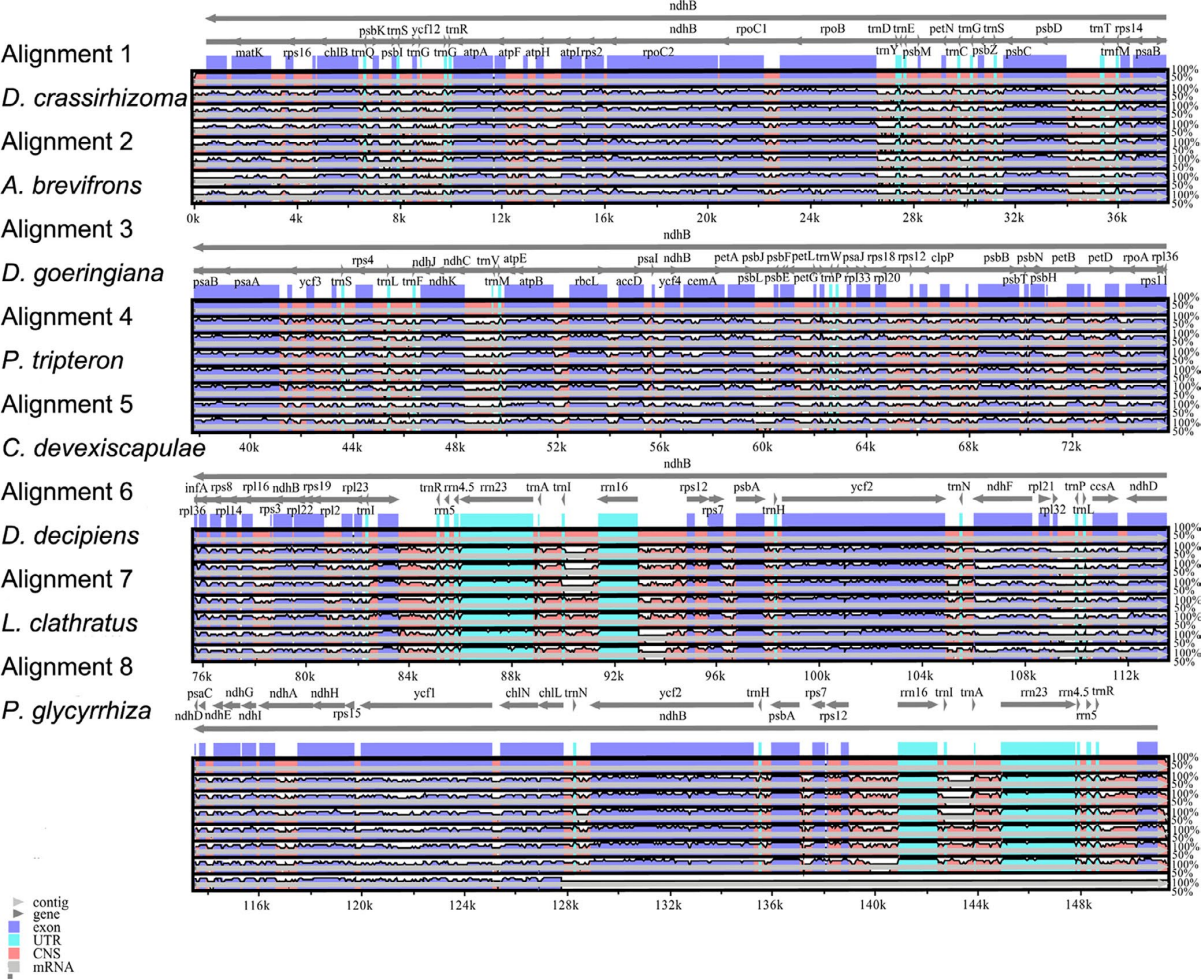
The sequence alignment of eight fern species. Gray arrows above the alignment indicate the orientation of genes. Purples, blue, and pink bars represent exons, introns and ncRNAs, and noncoding sequences, respectively. X‐axis represents the genome coordinate positions; Y‐axis represents the percent identify within 50%–100%. Dashed rectangles indicate highly divergent regions. Use *D. goeringiana* as the reference

The additional ferns *L. clathratus*, *D. decipiens*, *C. devexiscapulae*, and *P. glycyrrhiza* were selected for genome comparison analysis. Among the chloroplast genomes of these species, that of *L. clathratus* was the largest (156,998 bp), whereas that of *P. glycyrrhiza* was the smallest (129,221 bp). The chloroplast genomes ranged from 148,974 bp to 150,987 bp within *Dryopteris* genus, and the difference between the largest and smallest genomes was only 2,013 bp. However, this difference was 10‐fold in *Polypodium* (22,120 bp). Additionally, *rpoC2*, *rpoB*, *psbC*, *pasA*, *rbcL*, *ycf2*, *ycf1*, and *ndhB* were identified to be divergent among these chloroplast genomes (Figure 5). The sequences in the IR regions were more highly conserved than those in the LSC and SSC regions.

### 3.6 IR boundary contraction and expansion

**FIGURE 6:**
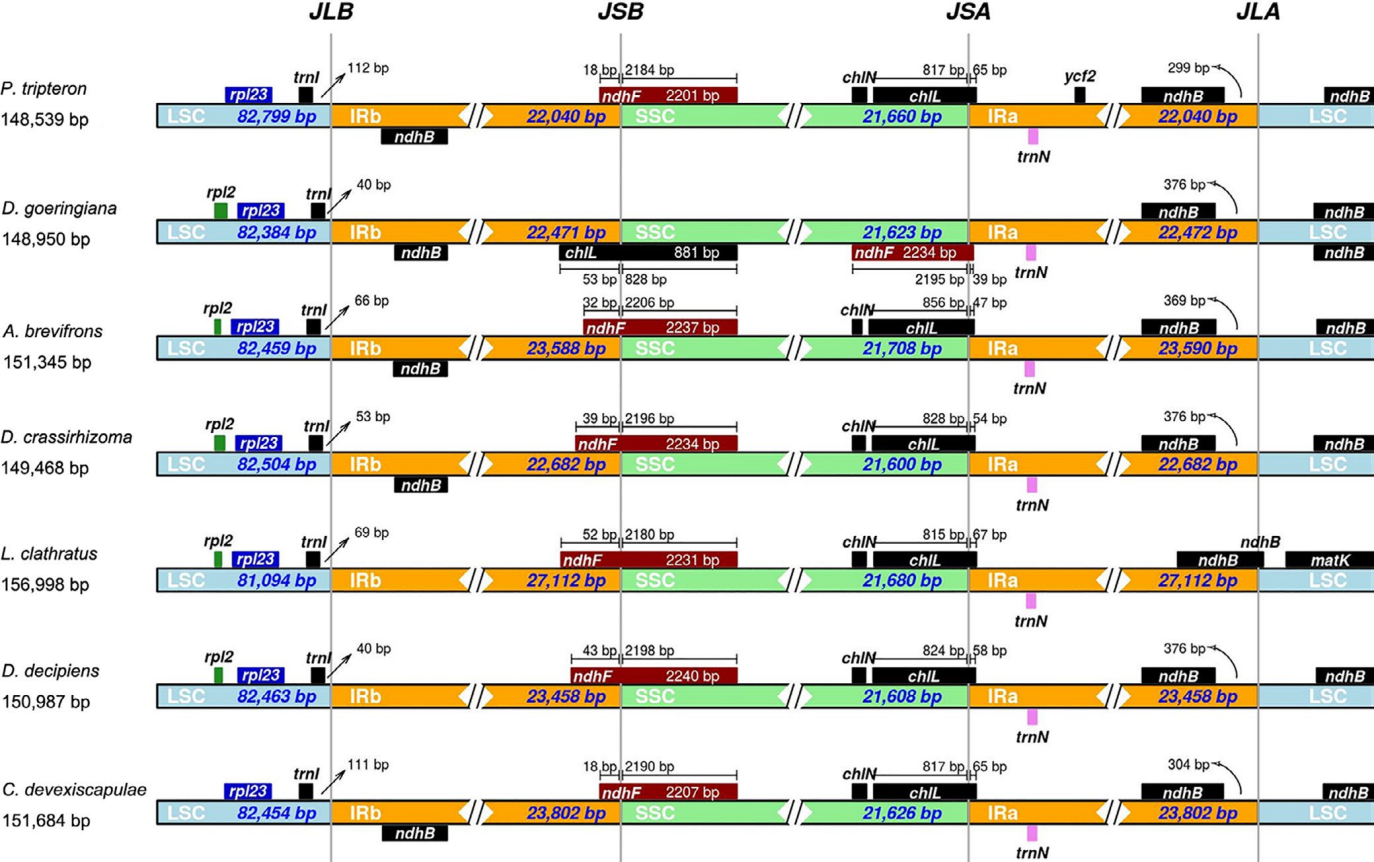
Comparison of the borders of LSC, SSC, and IR regions among the seven chloroplast genomes. The rectangular strips of each row represent a genome. The different colors represent different partitions. The black vertical line represents the boundary; the genes on both sides are indicated by small squares of different colors, the gene name is indicated on it, the gene in the forward chain is above; and the number represent the distant from gene and the boundary. Use *D. goeringiana* as the reference. LSC, large single copy. SSC, small single copy. IR, inverted repeat regions

IR boundary expansion and contraction were the main reasons for the differences in genome size, although IR regions were more highly conserved than LSC and SSC regions. The genes *trnI*, *ndhF*, *chlL*, and *ndhB* were located at LSC/IRb, IRb/SSC, SSC/IRa, and IRa/LSC boundaries, respectively (Figure 6). *trnI* was located in the LSC region, 40–112 bp away from the LSC/IRb boundary. *ndhF* crossed the IRb/SSC boundary, with 2,201–2,240 bp within the SSC region, except *D. goeringiana*. *chlL* crossed the SSC/IRa boundary, with 47–67 bp within the IRa region, except *D. goeringiana*. Notably, *D. goeringiana* presented an opposed gene order in the IRb/SSC and SSC/IRa regions compared with the other ferns, with *chlL* crossing the IRb/SSC region and *ndhF* crossing the SSC/IRa region. *ndhB* was located in the LSC region, 299–376 bp from the IRa/LSC boundary. However, *ndhB* extended into the LSC region of *L. clathratus*.

### 3.7 Phylogenetic analysis

**FIGURE 7:**
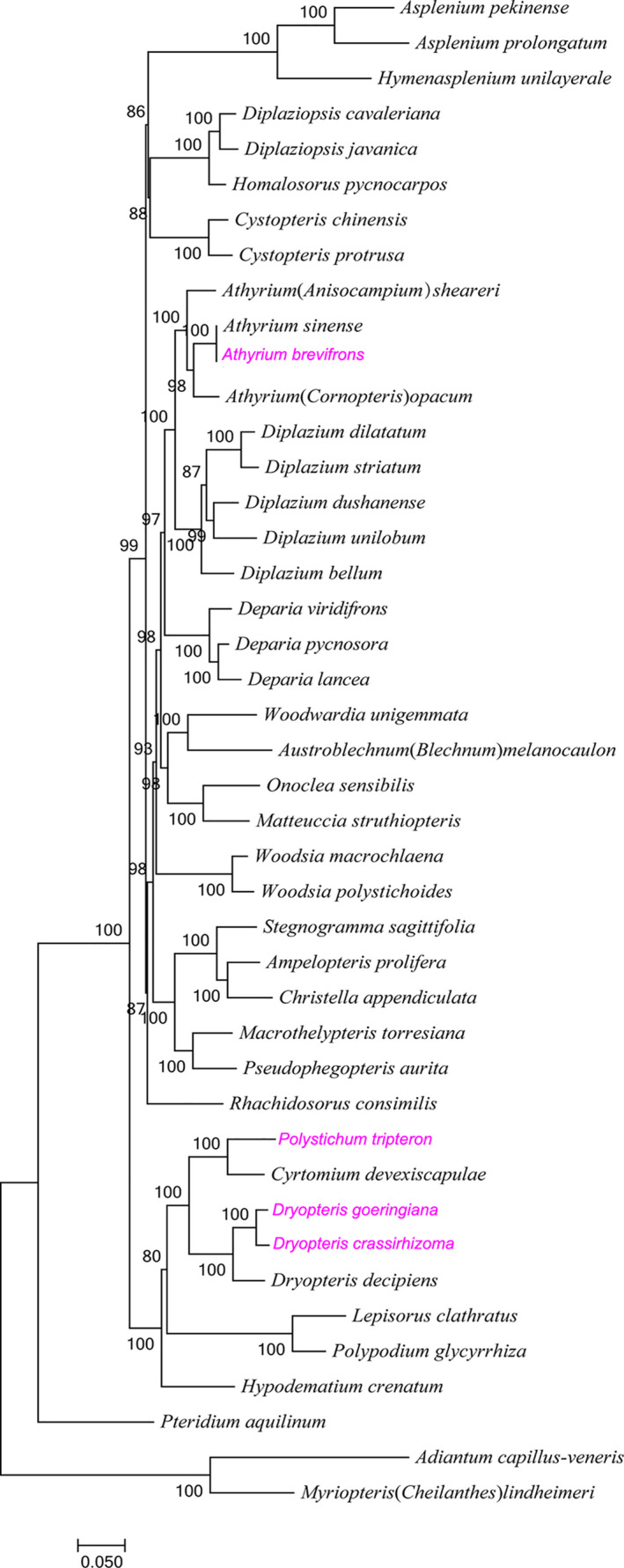
Molecular phylogenetic tree on 43 fern species. The tree was constructed using maximum‐likelihood algorithm and the general time‐reversible (GTR)+I + G + G model. The species studied in the present study was colored with pink

The phylogenetic tree (Figure 7) shows that 35 nodes had support values of > 90%, and 27 nodes had support values of 100%. The fern species in the same genus were clustered together to a certain degree. *D. crassirhizoma* and *D. goeringiana* were clustered and were shown to have a close relationship with *D. decipiens*. *P. tripteron* was identified as a sister species of *C. devexiscapulae*. Notably, *A. brevifrons* formed a single clade with *A. sinense* instead of *P. glycyrrhiza* (Figure 7).

## 4 DISCUSSION

The chloroplast genome provides information that is valuable for species identification, population genetics, plant phylogenetics, and genetic engineering. In the present study, three previously unpublished and one published fern were sequenced and compared with other species. The chloroplast genomes, which were 148,539–151,341 bp in length in the present study, were within the limit of fern chloroplast genomes (131,760–181,684 bp) reported by Gao et al., (2018) and Ruiz‐Ruano et al., 2018). The ferns in the present study had a typical four‐junction region structure (F. Liu & Pang, 2016; Mira Park et al., 2018). Variables were usually present in the LCS and SSC regions, and expansion and contraction were noted in the IR region (Asaf et al., 2017). Gao et al., (2018) and Lu et al., (2015) reported that intergenic sequences were extended, but overlapping genes were reduced in fern chloroplast genomes. Thus, sequence utilization is more specific, and genes are more independent. In the present study, we noted the absence of some genes in the ferns (*trnI‐GAU* was absent in *P. tripteron*), and that tRNA genes were more diverse than protein‐coding and rRNA genes, which possibly play an essential role in fern evolution.

Other factors that may promote evolution are introns within genes, boundary divergence, mutations, SSRs, and RNA editing events. Genes are interrupted by introns in the major groups of organisms. One‐intron genes vary among species, whereas *clpP*, *rps12*, and *ycf3* have been found to be two‐intron genes (Brouard et al., 2016; S. Liu et al., 2017; Ting Wang et al.,.., 2018); these findings were consistent with our observations, except for *P. tripteron*. One additional gene, *matK*, contained two introns in *P. tripteron*. *matK* is a useful biomarker for phylogenetic analysis in plant classification because its sequence evolution is faster than that of other chloroplast genes (Selvaraj et al., 2008). Notably, the two‐intron gene *matK* and the absence of *trnI‐GAU* may provide valuable evidence regarding molecular evolution of *P. tripteron*.

Although highly conserved, the expansion and contraction of IR regions are responsible for variations in chloroplast genome size and rearrangement (Raubeson et al., 2007; Yang et al., 2010), thereby promoting genomic evolution (Daniell et al., 2016; Logacheva et al., 2017). *D. fragrans* has a genome loss of 4,033 bp in the IR region, resulting in a longer SSC region and shorter IRs regions than those in *D. crassirhizoma* and *D. goeringiana* (Gao et al., 2018). Moreover, *D. fragrans* was found to have dispersed gene distribution and extended sequence lengths caused by more intergenic sequences. In the present study, *chlL* and *ndhF* were located at the SSC/IRa and IRb/SSC boundaries and extended into the IR regions with different lengths. All these ferns exhibited relatively similar IR boundary characteristics, except *D. goeringiana*, which presented an opposite gene order in the SSC/IRa and IRb/SSC junctions compared with another six fern species in the present study and another study (L. Xu et al., 2019). Further research is needed to determine whether and how this different gene order influences the evolution of *D. goeringiana*.

The nucleotide diversity analysis also demonstrated that the IR regions contained fewer variable loci than the SC regions. Additionally, genes with *Pi* values of > 0.20 were mainly located in the SC regions. None of the intron‐containing genes (*atpF*, *clpP*, *matK*, *ndhA*, *ndhB*, *petA*, *petB*, *petD*, *rpl16*, *rpl2*, *rpoC1*, *rps12*, *rps16*, and *ycf3*) had a *Pi* value of > 0.20, except *rpl16*. Intron‐containing genes are more highly conserved than exon‐containing genes only in the chloroplast genome. In other words, higher variability was found in exon‐containing genes, which provided more valuable information for species evolution. This result was consistent with that in fern plastomes (R. Wei et al., 2017).

SSRs, which serve as valuable molecular genetic markers, are widely used for population genetics (Doorduin et al., 2011; He et al., 2012) and plant genotyping (Ai‐Hong et al., 2011; Jianhua et al., 2012). We can conclude from the present study that the number and types of SSRs are conserved within genera because *D. crassirhizoma* and *D. goeringiana*, which belong to the same genus, demonstrated high similarity in the number and types of SSRs. The types of SSRs differ widely among genera, such as that observed between *A. brevifrons* and *P. tripteron*. SSRs are usually composed of a higher number of A + T bases than G + C bases (Dai‐Yong Kuang et al., 2011), which was in agreement with our observations. However, *D. fragrans* has higher G + C content than A + T content in SSRs. Taking the environment into consideration, Gao *et al*. speculated that the high G + C content in SSRs might help *D. fragrans* cope with substantial temperature differences (Gao et al., 2018). Vast differences in SSRs were noted among *D. fragrans*, *D. crassirhizoma*, and *D. goeringiana*, which belong to the same genus. We can speculate that the types of SSRs types are associated more with the surrounding environment than with the genus. This could explain the considerable differences among SSRs within the same species. The G + C content was similar among the ferns in the present study (42.40%–43.76%). Ferns have higher G + C content (41.49 ± 3.27) than gymnosperm (37.87 ± 1.56), angiosperm (37.71 ± 1.10), bryophyte (33.12 ± 4.16), and green algae (32.47 ± 6.07) (Kwon et al., 2020). The high G + C content of ferns helps them to survive in more environments than other plants.

The unparalleled G + C content might be explained by the high level of RNA editing in the organelles (Smith, 2009). It has been reported that fern and hornwort chloroplast genomes have evolved a higher number of RNA editing events than spermatophyte chloroplast genomes, in which only 30–40 RNA editing sites are typically present (Masanori et al., 2003; P. G. Wolf et al., 2004). In the present study, most editing events were C‐to‐U conversions, which was consistent with that reported in *D. fragrans* (Gao et al., 2018). It has been reported that the high number of C‐to‐U conversions developed in the early stages of vascular plant evolution (Koichiro et al., 2008). We can conclude that C‐to‐U RNA editing events might be essential for the rapid evolution of ferns.

Chloroplast genome data are valuable for resolving species definitions because organelle‐based “barcodes” can be established for certain species and then applied to reveal interspecies phylogenetic relationships (Jun‐Bo Yang et al.,.., 2013). The phylogenetic relationships of *A. brevifrons*, *D. goeringiana*, and *P. tripteron* have been rarely studied before chloroplast genome data became available. This study evaluated the chloroplast genomes of 43 fern species, almost all the ferns reported thus far, in the phylogenetic analysis. We found that *D. crassirhizoma* and *D. goeringiana* were closely related to *D. decipiens*. *P. tripteron* was identified as a sister species of *C. devexiscapulae*. Interestingly, *D. decipiens* and *C. devexiscapulae* were found to be clustered into one branch in a study by Wei *et al*. (R. Wei et al., 2017). These two species were reported to be closely related to *D. crassirhizoma* by Xu *et al*. (L. Xu et al., 2019). Based on the results of the present study, we can speculate that *D. crassirhizoma*, *D. goeringiana*, *D. decipiens*, *P. tripteron*, and *C. devexiscapulae* are closely related.

## 5 CONCLUSIONS

The complete chloroplast genomes of *D. goeringiana*, *A. brevifrons*, and *D. crassirhizoma* were sequenced for the first time. We also demonstrated that ferns have a higher G + C content and a higher number of C‐to‐U RNA editing events than other plants. The genomic characteristics and variations provide valuable information for understanding the evolution and phylogeny of ferns.

## ETHICS STATEMENT

None of the species are endangered, protected, or personally owned. This research was authorized by the Institute of Natural Resources and Ecology.

## CONFLICT OF INTEREST

We declare that we have no conflict of interest.

## AUTHOR CONTRIBUTION

**Ruifeng Fan:** Conceptualization (equal); Funding acquisition (lead); Writing‐original draft (lead); Writing‐review & editing (equal). **Wei Ma:** Methodology (lead); Visualization (equal); Writing‐review & editing (supporting). **Shilei Liu:** Software (lead); Writing‐review & editing (supporting). **Qingyang Huang:** Conceptualization (equal); Funding acquisition (supporting); Resources (lead); Writing‐review & editing (equal).

## Supporting information

Table S1

Table S2

## Data Availability

The sequences of the complete chloroplast genomes of *D. crassirhizoma*, *A. brevifrons*, *D. goeringiana*, and *P. tripteron* were deposited in GenBank (https://www.ncbi.nlm.nih.gov/genbank/) and under the accession numbers of MN712463, MN712464, MN712465, and MN712466, respectively.
