# Supplementary material for: Integrated analysis of three newly sequenced fern chloroplast genomes: Genome structure and comparative analysis
Source: Ecol Evol. 2021 Mar 18;11(9):4550–63. doi: 10.1002/ece3.7350 (PMC8093657; doi:10.1002/ece3.7350)
Supplement: Supplementary file 1 — Table S1 [file ECE3-11-4550-s002.docx]

Table S1. The detailed information of SSRs in four species.

| Repeats | 3 | 4 | 5 | 6 | 7 | 8 | 9 | 10 | 11 | 12 | 13 | 14 | 15 | 16 | 17 | 18 | 19 | 20 | 21 | total |
| --- | --- | --- | --- | --- | --- | --- | --- | --- | --- | --- | --- | --- | --- | --- | --- | --- | --- | --- | --- | --- |
| *P.tripteron* | | | | | | | | | | | | | | | | | | | | |
| A | - | - | - | - | - | 11 | 8 | 1 | 1 | 2 |  |  |  |  |  |  |  |  |  | 23 |
| C | - | - | - | - | - | 3 | 4 | 3 | 3 | 5 | 2 | 1 | 1 | 1 | 1 | 1 |  |  |  | 25 |
| G | - | - | - | - | - |  |  | 2 | 4 | 4 | 1 | 1 |  | 1 | 1 |  |  |  | 1 | 15 |
| T | - | - | - | - | - | 8 | 9 | 8 | 1 | 1 |  |  |  |  |  |  |  |  |  | 27 |
| AT | - | - | 2 | 2 | 1 |  |  | 1 |  |  |  |  |  |  |  |  |  |  |  | 6 |
| TC | - | - | 1 |  |  |  |  |  |  |  |  |  |  |  |  |  |  |  |  | 1 |
| TG | - | - | 1 |  |  |  |  |  |  |  |  |  |  |  |  |  |  |  |  | 1 |
| AGA | - | 3 |  |  |  |  |  |  |  |  |  |  |  |  |  |  |  |  |  | 3 |
| AATA | 1 |  |  |  |  |  |  |  |  |  |  |  |  |  |  |  |  |  |  | 1 |
| AATT | 1 |  |  |  |  |  |  |  |  |  |  |  |  |  |  |  |  |  |  | 1 |
| ATAA | 1 |  |  |  |  |  |  |  |  |  |  |  |  |  |  |  |  |  |  | 1 |
| ATAG | 1 |  |  |  |  |  |  |  |  |  |  |  |  |  |  |  |  |  |  | 1 |
| GATA | 1 |  |  |  |  |  |  |  |  |  |  |  |  |  |  |  |  |  |  | 1 |
| TCTA | 1 |  |  |  |  |  |  |  |  |  |  |  |  |  |  |  |  |  |  | 1 |
| ATATA | 1 |  |  |  |  |  |  |  |  |  |  |  |  |  |  |  |  |  |  | 1 |
| *D.crassirhizoma* | | | | | | | | | | | | | | | | | | | | |
| A | - | - | - | - | - | 9 | 3 | 3 |  | 1 | 2 |  |  |  |  |  |  |  |  | 18 |
| C | - | - | - | - | - | 5 | 1 |  | 2 | 3 | 1 | 2 |  | 2 | 1 |  |  |  |  | 17 |
| G | - | - | - | - | - | 4 |  |  | 2 | 2 | 1 |  |  | 1 | 2 |  |  |  |  | 12 |
| T | - | - | - | - | - | 3 | 2 | 3 | 1 |  |  |  |  |  |  |  |  |  |  | 9 |
| AT | - | - | 1 | 1 | 1 |  | 1 | 2 |  |  |  |  |  |  |  |  |  |  |  | 6 |
| TA | - | - |  |  | 1 | 1 |  |  |  |  |  |  |  |  |  |  |  |  |  | 2 |
| TG | - | - | 1 |  |  |  |  |  |  |  |  |  |  |  |  |  |  |  |  | 1 |
| TCC | - | 1 |  |  |  |  |  |  |  |  |  |  |  |  |  |  |  |  |  | 1 |
| TTC | - | 1 |  |  |  |  |  |  |  |  |  |  |  |  |  |  |  |  |  | 1 |
| AGAT | 1 |  |  |  |  |  |  |  |  |  |  |  |  |  |  |  |  |  |  | 1 |
| ATAA | 2 |  |  |  |  |  |  |  |  |  |  |  |  |  |  |  |  |  |  | 2 |
| ATAG |  | 1 |  |  |  |  |  |  |  |  |  |  |  |  |  |  |  |  |  | 1 |
| ATCT | 1 |  |  |  |  |  |  |  |  |  |  |  |  |  |  |  |  |  |  | 1 |
| CTAT | 1 |  |  |  |  |  |  |  |  |  |  |  |  |  |  |  |  |  |  | 1 |
| TTCT | 1 |  |  |  |  |  |  |  |  |  |  |  |  |  |  |  |  |  |  | 1 |
| TTTA | 1 |  |  |  |  |  |  |  |  |  |  |  |  |  |  |  |  |  |  | 1 |
| *A. brevifrons* | | | | | | | | | | | | | | | | | | | | |
| A | - | - | - | - | - | 7 | 5 | 10 | 3 |  |  |  |  |  |  |  |  |  |  | 25 |
| C | - | - | - | - | - | 8 | 3 | 1 | 3 | 1 | 1 | 1 | 1 | 1 | 1 |  |  |  |  | 21 |
| G | - | - | - | - | - | 5 | 1 | 4 | 4 | 1 | 1 | 2 | 2 |  |  |  |  |  |  | 20 |
| T | - | - | - | - | - | 8 | 1 | 7 | 1 |  |  |  |  |  |  |  |  |  |  | 17 |
| AT | - | - | 1 |  | 1 | 1 |  |  | 1 |  |  |  |  |  |  |  |  |  |  | 4 |
| CT | - | - | 2 |  |  |  |  |  |  |  |  |  |  |  |  |  |  |  |  | 2 |
| GA | - | - | 1 |  |  |  |  |  |  |  |  |  |  |  |  |  |  |  |  | 1 |
| TA | - | - | 1 | 1 |  |  |  |  |  |  |  |  |  |  |  |  |  |  |  | 2 |
| TC | - | - | 3 |  |  |  |  |  |  |  |  |  |  |  |  |  |  |  |  | 3 |
| CCG | - | 1 |  |  |  |  |  |  |  |  |  |  |  |  |  |  |  |  |  | 1 |
| GCG | - | 1 |  |  |  |  |  |  |  |  |  |  |  |  |  |  |  |  |  | 1 |
| AGAA | 1 |  |  |  |  |  |  |  |  |  |  |  |  |  |  |  |  |  |  | 1 |
| ATAG | 1 |  |  |  |  |  |  |  |  |  |  |  |  |  |  |  |  |  |  | 1 |
| ATTA | 1 |  |  |  |  |  |  |  |  |  |  |  |  |  |  | 1 |  |  |  |  |
| CTAT | 1 |  |  |  |  |  |  |  |  |  |  |  |  |  |  | 1 |  |  |  |  |
| GGTA | 1 |  |  |  |  |  |  |  |  |  |  |  |  |  |  | 1 |  |  |  |  |
| TATC | 3 |  |  |  |  |  |  |  |  |  |  |  |  |  |  | 3 |  |  |  |  |
| TTTC | 1 |  |  |  |  |  |  |  |  |  |  |  |  |  |  | 1 |  |  |  |  |
| CTTAT | 1 |  |  |  |  |  |  |  |  |  |  |  |  |  |  | 1 |  |  |  |  |
| TTTCT | 1 |  |  |  |  |  |  |  |  |  |  |  |  |  |  | 1 |  |  |  |  |
| *D.goeringiana* | | | | | | | | | | | | | | | | | | | | |
| A | - | - | - | - | - | 6 | 3 | 5 | 1 |  |  |  |  |  |  |  |  |  |  | 15 |
| C | - | - | - | - | - | 3 | 3 | 4 |  | 2 | 2 |  | 1 |  |  |  |  |  |  | 15 |
| G | - | - | - | - | - | 3 | 3 | 2 | 1 |  | 1 | 1 |  |  |  |  |  |  |  | 11 |
| T | - | - | - | - | - | 6 | 4 | 3 | 1 |  |  |  |  |  |  |  |  |  |  | 14 |
| AT | - | - | 1 |  |  | 1 | 1 | 1 |  |  |  |  |  |  |  |  |  |  |  | 4 |
| TA | - | - |  | 1 | 1 | 1 |  |  |  |  |  |  |  |  |  |  |  |  |  | 3 |
| TG | - | - | 1 |  |  |  |  |  |  |  |  |  |  |  |  |  |  |  |  | 1 |
| TCC | - | 1 |  |  |  |  |  |  |  |  |  |  |  |  |  |  |  |  |  | 1 |
| TTC | - | 1 |  |  |  |  |  |  |  |  |  |  |  |  |  |  |  |  |  | 1 |
| AGAT | 1 |  |  |  |  |  |  |  |  |  |  |  |  |  |  |  |  |  |  | 1 |
| ATAG |  | 1 |  |  |  |  |  |  |  |  |  |  |  |  |  |  |  |  |  | 1 |
| ATCT | 1 |  |  |  |  |  |  |  |  |  |  |  |  |  |  |  |  |  |  | 1 |
| CTAT | 1 |  |  |  |  |  |  |  |  |  |  |  |  |  |  |  |  |  |  | 1 |
