# Supplementary material for: Integrated analysis of three newly sequenced fern chloroplast genomes: Genome structure and comparative analysis
Source: Ecol Evol. 2021 Mar 18;11(9):4550–63. doi: 10.1002/ece3.7350 (PMC8093657; doi:10.1002/ece3.7350)
Supplement: Supplementary file 2 — Table S2 [file ECE3-11-4550-s001.docx]

Table S2. The detailed information of Nucleotide diversity analysis.

| Gene | Length | S | Eta | Pi |
| --- | --- | --- | --- | --- |
| trnM-CAU | 64 | 22 | 23 | 0.190104 |
| trnE-UUC | 67 | 22 | 25 | 0.19403 |
| psbZ | 185 | 68 | 74 | 0.202703 |
| trnN-GUU | 72 | 27 | 30 | 0.208333 |
| trnI-CAU | 74 | 29 | 33 | 0.216216 |
| rpl21 | 356 | 143 | 147 | 0.219101 |
| psbM | 98 | 39 | 43 | 0.226191 |
| rpl32 | 164 | 75 | 78 | 0.253049 |
| trnV-UAC | 37 | 18 | 18 | 0.256757 |
| rpl16-CDS1 | 9 | 5 | 5 | 0.277778 |

Pi, polymorphism information.
